# Exosomal miR-141 promotes tumor angiogenesis via KLF12 in small cell lung cancer

**DOI:** 10.1186/s13046-020-01680-1

**Published:** 2020-09-21

**Authors:** Shuangshuang Mao, Zhiliang Lu, Sufei Zheng, Hao Zhang, Guochao Zhang, Feng Wang, Jianbing Huang, Yuanyuan Lei, Xinfeng Wang, Chengming Liu, Nan Sun, Jie He

**Affiliations:** Department of Thoracic Surgery, National Cancer Center/National Clinical Research Center for Cancer/Cancer Hospital, Chinese Academy of Medical Sciences and Peking Union Medical College, Beijing, 100021 China

**Keywords:** SCLC, Exosomes, miR-141, HUVEC, Angiogenesis

## Abstract

**Background:**

Angiogenesis, a basic requirement for tumor cell survival, is considered to be a malignant characteristic of small cell lung cancer (SCLC) and is closely related to the poor outcomes of SCLC patients. miR-141 has been found to play pro- and antiangiogenic roles in different cancers, but its role in SCLC angiogenesis has never been explored.

**Methods:**

Total RNA was isolated from plasm exosomes and serum of SCLC patients to examine the expression of miR-141 by qRT-PCR. Cell proliferation, invasion, migration, tube formation assay, aortic ring assay and mouse tumor model were used to investigate the effect of exosomal miR-141 in angiogenesis in vitro and in vivo. Dual-luciferase assay was conducted to explore the target gene of miR-141.

**Results:**

Circulating miR-141 was upregulated in samples from 122 SCLC patients compared with those from normal volunteers and that the increase in miR-141 was significantly associated with advanced TNM stages, implying the potential oncogenic role of miR-141 in SCLC malignancy. In vitro, miR-141 that was packaged into SCLC cell-secreted exosomes and delivered to human umbilical vein vascular endothelial cells (HUVECs) via exosomes facilitated HUVEC proliferation, invasion, migration and tube formation and promoted microvessel sprouting from mouse aortic rings. Matrigel plug assays demonstrated that SCLC cell-derived exosomal miR-141 induced neoangiogenesis in vivo. Furthermore, mouse subcutaneous tumor nodules that were developed from miR-141-overexpressing SCLC cells had a higher microvessel density (MVD) and grew faster than those developed from negative control cells. KLF12 was found to be the direct target gene of miR-141 and that the proangiogenic effect of miR-141 on HUVECs was abrogated by KLF12 overexpression.

**Conclusions:**

Our results demonstrate the specific function of the exosomal miR-141/KLF12 pathway in SCLC angiogenesis for the first time and provide potential novel targets for antiangiogenic therapies for SCLC patients.

## Background

Lung cancer, one of the most common malignant tumors, is the leading cause of cancer-related deaths among both men and women [1, 2], with small cell lung cancer (SCLC) accounting for about 15% of all lung cancers [3]. SCLC is a highly aggressive neuroendocrine tumor characterized by uncontrolled fast tumor growth, abundant neoangiogenesis, early metastasis, drug resistance and early disease relapse, which together lead to extremely poor outcomes for SCLC patients [3, 4]. Tumor angiogenesis facilitates tumor growth, survival and metastasis and is a hallmark of cancer [5]. Tumor-associated neovasculatures have been found to play pivotal roles in the development and progression of SCLC and are related to the poor prognosis of SCLC patients [6, 7]. A number of anti-angiogenetic agents, such as bevacizumab and sorafenib, have been approved by the Food and Drug Administration (FDA) for the treatment of non-small cell lung cancer (NSCLC) and other solid tumors and have obtained promising results [8–11]. However, clinical trials containing antiangiogenetic drugs in the therapy of SCLC patients showed limited effects [12–15] and the traditional therapy for SCLC patients have not been advanced for about three decades [3]. Thus, further exploration of the molecular mechanisms of SCLC-associated angiogenesis might help to clarify the complex angiogenetic processes in SCLC microenvironment which might broaden our knowledge about SCLC malignancy and have potential value in promoting the development of effective antiangiogenic therapeutics in SCLC patients.

MicroRNAs (miRNAs), post-transcriptional regulators of gene expression, are a group of endogenous small noncoding RNAs that have approximately 23 nucleotides. miRNAs mediate the degradation or translational inhibition of their downstream messenger RNAs (mRNAs) by binding to the seeding sequences of the 3′ untranslated regions (3’UTRs) of these mRNAs [16]. miRNAs have been shown to be up- or downregulated in various tumor types [17, 18], and dysregulated miRNA expression has been widely demonstrated to affect multiple tumor biological processes, including tumor angiogenesis [19–21].

miR-141 is one of members belonging to the miR-200 family, which is a group of miRNAs documented to regulate the epithelial to mesenchymal transition (EMT) of tumors by regulating two EMT-associated genes, ZEB1 and ZEB2 [22, 23]. However, the effects of miR-141 on tumor angiogenesis remain unclear, as conflicting evidence suggests a dual role for miR-141 in the neovascularization of different cancer types [24–27]. For example, in basal-like breast cancers, miR-141 was found to reduce microvessel density (MVD) and inhibit tumor growth by targeting IL-8 and CXCL1 [25]. In contrast, in non-small cell lung cancers, tumor tissues with higher expression of miR-141 showed significantly more tumor-associated blood vessels than those with lower miR-141 expression [27]. The pro- and antiangiogenic effects of miR-141 are complicated and confusing, and the specific function of miR-141 in SCLC angiogenesis has never been explored.

Exosomes, small extracellular vesicles containing numerous miRNAs, protect RNAs from degradation by circulating RNA enzymes and transfer biological information between cells and microenvironments [28]. The realization of the effects of miRNAs on tumor angiogenesis mainly depends on transport and protection by these vesicles [29–31]. Thus, in the present study, we focused on exosomal miR-141 to study its effect on SCLC angiogenesis. We found that miR-141 could be transferred from SCLC cells to human umbilical vein vascular endothelial cells (HUVECs) via SCLC cell-secreted exosomes and that internalized miR-141 promoted HUVEC proliferation, migration, tube formation and new blood vessel formation in vitro and in vivo. KLF12 was demonstrated to be directly downregulated by miR-141, and in HUVECs, the effects of KLF12 knockdown were similar to those of miR-141 overexpression. Additionally, miR-141 was confirmed to be significantly upregulated in both the plasma and serum of SCLC patients and was related to patient TNM stages. Taken together, our research reveals novel details elaborating the mechanism of exosomal miR-141 in SCLC angiogenesis and offers a promising clue for identifying effective antiangiogenic targets to improve the prognosis of SCLC patients.

## Methods

### SCLC patient specimens

Both plasma (*n* = 77) and serum samples (*n* = 101) from SCLC patients and normal volunteers were collected from the National Cancer Center/National Clinical Research Center for Cancer/Cancer Hospital. All SCLC patients were pathologically confirmed to have SCLC by surgery or tissue aspiration biopsy, and all blood samples were collected before any antitumor therapies. Informed consent was acquired from every participant, and approval of the experimental protocol was obtained from the medical ethics committee of the National Cancer Center/National Clinical Research Center for Cancer/Cancer Hospital. Detailed information about the clinical and pathological features of all individuals is listed in Supplementary Table 1.

### Exosome isolation, characterization and quantification

Exosomes from the SCLC cell culture medium (CM) were isolated by ultracentrifugation. Briefly, cells were refreshed with CM containing 10% exosome-removed fetal bovine serum (FBS) (ultracentrifuged at 110,000×g at 4 °C overnight) after washing with phosphate-buffered saline (PBS) twice. The CM was collected after the cells were cultured for 48 h. The obtained CM was processed for differential centrifugation at 500×g for 10 min, 2000×g for 10 min and 10,000×g for 30 min successively to remove dead cells, cell debris and large vesicles. The supernatant was then ultracentrifuged at 110,000×g for 70 min to collect exosome pellet followed by washing pellet with PBS once. All centrifugation procedures were conducted under the condition of 4 °C. The obtained exosomes were finally resuspended and preserved in PBS. The BCA assay was used to detect the protein concentration of exosomes. 15 μg of exosomes were used to stimulate 1 × 10^5^ HUVECs. To isolate exosomes from plasma, we used the Total Exosome Isolation (from plasma) Kit (Invitrogen, cat. No. 4484450) to precipitate exosomes according to the manufacturer’s instructions.

A total of 10 μl of exosomes were used for sample preparation to observe exosomes under a transmission electron microscope (JEOL-JEM1400, Tokyo, Japan). Briefly, after incubation at room temperature for 10 min, exosomes on a copper mesh were washed with sterile distilled water followed by incubation with uranyl-oxalate solution for 1 min. The copper mesh was then dried for 2 min under incandescent light and observed under a microscope. The exosome suspension was diluted by PBS to examine the size and quantity of exosomes under a ZetaView PMX 110 (Particle Metrix, Meerbusch, Germany). Exosome-specific proteins, namely, CD9 and TSG101, were detected by western blot.

### RNA oligonucleotides, plasmids and virus

The following were synthesized by GenePharma (Shanghai, China): both the mimics and inhibitors of miR-141, their respective negative controls and Cy3-labeled mimics of miR-141 (miR-141, 5′-UAACACUGUCUGGUAAAGAUGG-3′; negative control (NC), 5′-UUCUCCGAACGUGUCACGUTT-3′; Anti-miR-141, 5′-CCAUCUUUACCAGACAGUGUUA-3′; Anti-NC, 5′-CAGUACUUUUGUGUAGUACAA-3′). Small interfering RNAs (siRNAs) of KLF12 together with their negative control (siRNA-1, 5′-GGACUCGUUAUCUGUAGAUTT-3′; siRNA-2, 5′-GCACAUUAUCCAUCCCGUATT-3′; siRNA-NC, 5′-UUCUCCGAACGUGUCACGUTT-3′) were synthesized by Sangon Biotech (Shanghai, China). Plasmids for KLF12 overexpression were purchased from Vigene Biosciences (Shandong, China).

To construct the dual-luciferase reporter vectors of KLF12, three 3′ UTR fragments of KLF12 containing the predicted seeding sequences or the mutant ones were inserted into PGL3.0 vectors that expressed firefly luciferase. The plasmid expressing Renilla luciferase was used as the internal reference.

The lentiviral vectors expressing pri-miR-141 (OE-miR-141) or the control (OE-NC), together with three lentiviral packaging vectors were cotransfected into 293 T cells, and the lentivirus was collected after 48–72 h to infect SCLC cells. All transfection assays were carried out using Lipofectamine RNAiMAX Transfection Reagent (Invitrogen, cat. No. 13778150) or Lipofectamine 3000 Transfection Reagent (Invitrogen, cat. No. L3000015) following the manufacturer’s instructions.

### Cell culture and subline construction

SCLC cell lines (H446 and H1048) obtained from the American Type Culture Collection (ATCC) were maintained in the recommended CM in an incubator containing 5% CO_2_ at 37 °C. Dulbecco’s modified Eagle’s medium (DMEM, Corning) containing 10% FBS (Gibco) and 1% penicillin and streptomycin (Gibco) was used to culture HUVECs and EAhy.926 cells. To develop SCLC sublines that stably overexpressed miR-141, SCLC cells were screened with puromycin after lentivirus infection for half a month and then maintained in CM with low-dose puromycin.

### Exosome processing and internalization

To visualize the internalization of exosomal miR-141 in HUVECs, exosomes isolated from H446 cells, which were transfected with Cy3-labeled mimics of miR-141 for 48 h, were labeled with PKH67 and incubated with HUVECs. After 12 h, exosome-treated HUVECs were photographed under a fluorescence microscope.

### RNA isolation, PCR and western blotting

TRIzol reagent was utilized to extract total RNA from cells, patient serum and plasma exosomes, with cel-miR-39 added to serum or exosome solutions before chloroform. Cel-miR-39 was used as the external reference for the PCR analysis of miRNA in serum or exosomes, while RNU6 was used as the internal reference for the detection of miRNA in cells. The primers of miRNAs or mRNAs used in the present study were listed as follows: miR-141 (Forward, 5′-CGCTAACACTGTCTGGTAAAGATGG-3′); cel-miR-39 (Forward, 5′-TCACCGGGTGTAAATCAGCTTG-3′); RNU6 (Forward, 5′-CTCGCTTCGGCAGCACA-3′); KLF12 (Forward, 5′-CCTTTCCATAGCCAGAGCAGTAC-3′; Reverse, 5′-CTGGCGTCTTGTGCTCTCAATAC-3′); and GAPDH (Forward, 5′-GTCTCCTCTGACTTCAACAGCG-3′; Reverse, 5′-ACCACCCTGTTGCTGTAGCCAA-3′).

RIPA lysis reagent was used to isolate proteins from cells or exosomes, and the concentrations of proteins were measured using BCA assay method. Proteins with different molecular weights were subjected to sodium dodecyl sulfate–polyacrylamide gel electrophoresis (SDS-PAGE) followed by transferring onto PVDF membranes. The membranes containing separated proteins were washed and then incubated with the objective primary antibodies overnight at 4 °C. The appropriate HRP-conjugated secondary antibodies were incubated with the membranes the next day to detect the chemiluminescence signals. The primary antibodies used in the present study included KLF12 (Abcam, ab129459), GAPDH (CST, #5174), CD9 (Abcam, ab92726), and TSG101 (Abcam, ab83).

### Cell proliferation, invasion and migration assays

For cell proliferation assay, 1500 HUVECs or 2500 EAhy.926 cells were added into 96-well plates per well, Cell Counting Kit 8 (CCK8, Dojindo) was used to detect the cell viability every day for 3–4 days.

Transwell inserts (Corning) were utilized to evaluate the invasion and migration of vascular endothelial cells. Cells (6 × 10^4^ or 3 × 10^4^) were resuspended in FBS-depleted CM and placed in the upper chambers of 24-well inserts coated with or without Matrigel. After twenty hours, cells remaining on the insert upper surfaces, namely, that did not pass through the pores, were wiped off. Then, cells on the insert lower surfaces were carefully fixed with methyl alcohol followed by staining with Giemsa, and the invaded or migrated cells were counted under a microscope.

### Tube formation and aortic ring assay

For tube formation assays, 50 μl of Matrigel was added to each well of a precooled 96-well plate and incubated at 37 °C for 1 h. HUVECs or EAhy.926 cells were trypsinized, and their concentrations were adjusted to 2 × 10^5^/ml or 5 × 10^5^/ml, respectively. A total of 100 μl of cell suspension was added to each well of the Matrigel-precoated 96-well plate, and the tubes that formed were imaged and counted under a microscope.

For aortic ring assays, the thoracic aortas of 8-week-old C57BL/6 mice were processed according to a previously published protocol [32]. Briefly, the dissected thoracic aorta was first flushed gently with Opti-MEM (Gibco) through its lumen to remove residual blood, and the aorta was then cut into 0.5-mm rings. The obtained aortic rings were incubated in a starvation medium, namely, Opti-MEM, at 37 °C overnight to equilibrate their response to stimulation. Nucleotide transfections or exosome treatments were conducted at the same time as starvation of the aortic rings. Afterward, aortic rings were embedded in collagen (collagen type I, rat tail, Millipore) and supplemented with Opti-MEM CM containing 2.5% FBS and 30 ng/ml VEGF (Peprotech, cat. No. 450–32-2). Microvessels that sprouted from the aortic rings were observed under a microscope.

### Luciferase reporter assay

HUVECs were cotransfected with reporter plasmids containing the 3’UTR of KLF12 and the Renilla luciferase vector, together with miR-141 mimics or the NC, using the Lipofectamine 3000 transfection reagent. Cell lysates were harvested 48 h later and added to 96-well plates, and luciferase activity was measured with a microplate reader.

### In vivo Matrigel plug assay

A total of 500 μl of growth factor-reduced Matrigel (Corning, cat. No. 356231) mixed with exosomes was injected into the right axillae of C57BL/6 mice (6 weeks old) subcutaneously. The plugs that had formed were removed ten days later, fixed with formalin and sliced for immunohistochemistry (IHC). The presence of the blood vessel-specific marker CD31 was assessed with CD31 antibody (Abcam, ab28364) to measure the density of vessels generated in the plug.

### Animal model

To evaluate whether miR-141 could promote SCLC tumor growth by inducing angiogenesis, 5 × 10^6^ H1048 cells or 1 × 10^7^ H446 cells stably overexpressing miR-141 were injected into BALB/c nude mice (female, 6 weeks of age) subcutaneously. Tumor growth was monitored two times per week. The tumor MVD was evaluated by IHC of CD31. Mouse plasma exosomes were isolated using the Total Exosome Isolation (from plasma) Kit, and exosomal miR-141 was measured by qRT-PCR.

### Statistical analysis

GraphPad Prism software was utilized to perform all analysis of experimental data, with the results presented as the mean ± SD. The statistical differences between two groups were detected by Student’s t-test, and one-way ANOVA was applied for three or more groups. Statistical results with *P < 0.05, **P < 0.01, ***P < 0.001, or ****P < 0.0001, were considered to be statistically significant.

## Results

### MiR-141 is aberrantly upregulated in the plasma and serum of SCLC patients

**Fig. 1:**
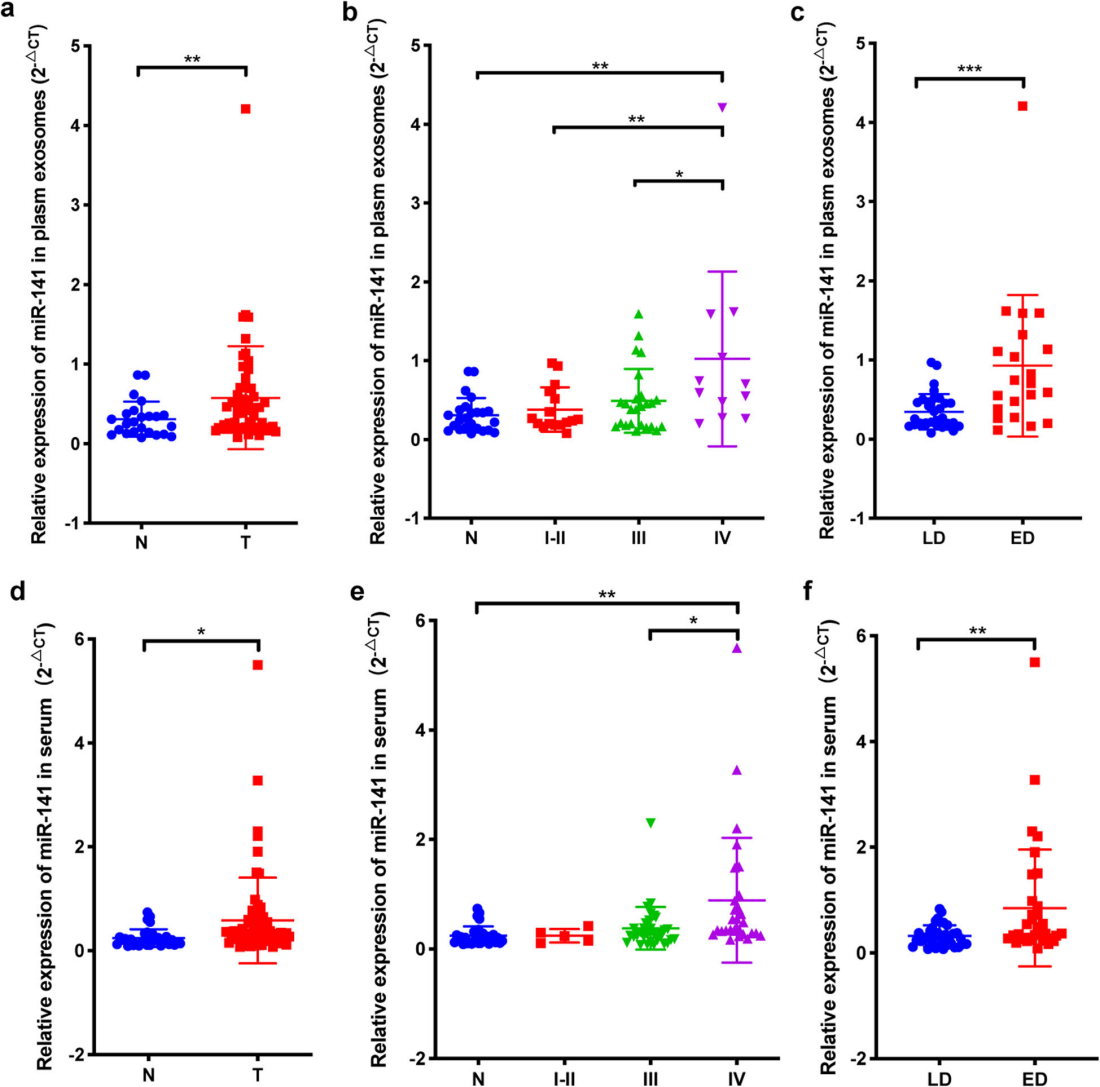
Circulating miR-141 is aberrantly upregulated in patients with SCLC. A. The relative expression of miR-141 was higher in plasma exosomes from SCLC patients than that in those from healthy volunteers. B. The level of plasma exosomal miR-141 was particularly high in stage IV SCLC patients. C. SCLC patients with extensive disease had a higher expression of miR-141 in plasma exosomes than whom with limited disease. D. The relative expression of miR-141 was higher in serum samples from SCLC patients than that in those from healthy volunteers. E. The expression of circulating miR-141 was particularly high in the serum of stage IV SCLC patients. F. SCLC patients with extensive disease had a higher expression of miR-141 in serum than whom with limited disease. N, normal; T, tumor; I, stage I; II, stage II; III, stage III; IV, stage IV; LD, limited disease; ED, extensive disease

To better understand the clinical importance of miR-141 in patients with SCLC, we measured the expression of miR-141 in both plasma and serum samples. A total of 77 plasma samples from 53 SCLC patients and 24 healthy volunteers were used for exosome isolation followed by RNA extraction and qRT-PCR analysis. We found that miR-141 levels in plasma exosomes were significantly higher in SCLC patients than in healthy controls (Fig. 1A). More importantly, the level of plasm exosomal miR-141 in 12 SCLC patients with stage IV disease was much higher than SCLC patients with stage I-III disease (Fig. 1B), SCLC patients with extensive disease also showed much higher levels of exosomal miR-141 than whom with limited disease (Fig. 1C). To further explore the level of circulating miR-141 in SCLC patients, another set of 101 serum samples from 69 SCLC patients and 32 healthy volunteers was used for total RNA isolation and detection. Consistent with the miR-141 levels in plasma exosomes, those of miR-141 in the serum of SCLC patients were aberrantly increased (Fig. 1D), especially in stage IV patients (Fig. 1E), and SCLC patients with extensive disease also showed much higher levels of circulating miR-141 in serum than whom with limited disease (Fig. 1F). Additionally, clinical correlation analysis demonstrated that higher miR-141 expression was related to larger tumor size, more lymph node metastasis, more distant metastasis and extensive stage (Table 1). As we know, tumor-associated vasculatures transport oxygen and nutrient to tumor cells to promote tumor cell survival and growth and provide opportunities for tumor cell metastasis, which indicated that angiogenesis was closely related to tumor development and progression [5]. Thus, our data implies the potential importance of miR-141 in SCLC-associated angiogenesis.

**Table 1:** Relationships between miR-141 expression and SCLC patient clinical characteristics

		Plasma exosomes (*n* = 53)			Serum (*n* = 69)		
		miR-141 low level	miR-141 high level	*P* value	miR-141 low level	miR-141 high level	P value
Sex	Female	12	7	0.1248	7	7	0.9516
	Male	14	20		27	28	
Age	< 60	11	17	0.1321	12	16	0.3782
	≥60	15	10		22	19	
T	T1-T2	17	8	0.0091**	17	11	0.1163
	T3-T4	9	19		17	24	
N	N0-N1	12	5	0.0312*	7	3	0.1563
	N2-N3	14	22		27	32	
M	M0	23	18	0.0581	25	15	0.0099**
	M1	3	9		9	20	
Stage	I-II	11	5	0.0593	4	1	0.1536
	III-IV	15	22		30	34	
Stage	LD	20	12	0.0244*	21	14	0.0706
	ED	6	15		13	21	

LD, limited disease; ED, extensive disease

miR-141 low- and high-level group were defined according to the median of miR-141 expression in the corresponding cohort of SCLC patients

### miR-141 in SCLC cell-secreted exosomes promotes HUVEC proliferation, migration, tube formation and angiogenesis in vitro

**Fig. 2:**
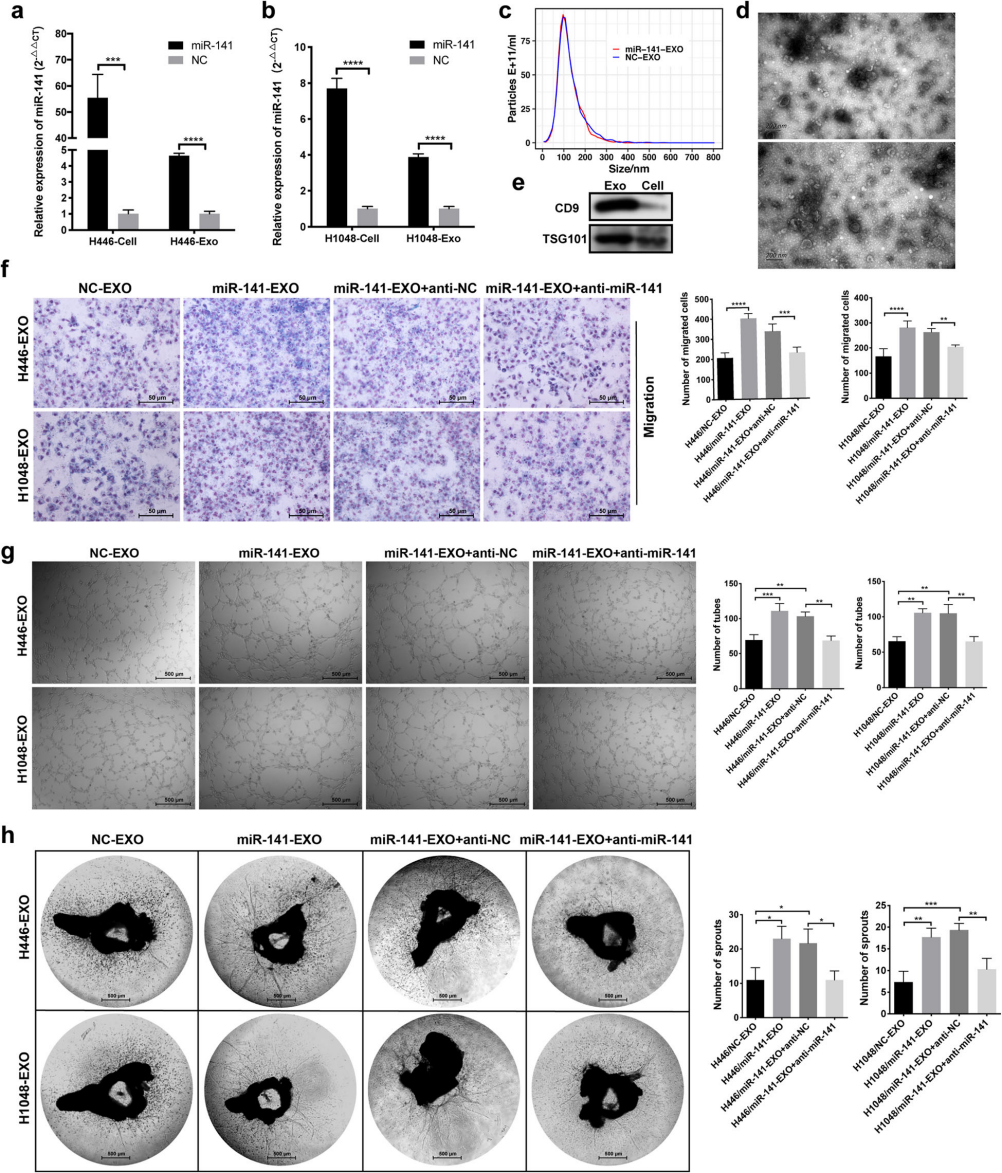
Exosomal miR-141 derived from SCLC cells promotes HUVEC migration and tube formation. A, B. After miR-141-mimic transfection, the relative expression of miR-141 was increased in H446 and H1048 cells and in their exosomes. C. The size and quantitation of exosomes isolated from miR-141- or NC-transfected H446 cells was examined by nanoparticle tracking analysis. D. TEM images of exosomes isolated from H446 cells. E. The level of CD9 and TSG101 was detected by western blot. F. Representative images of HUVECs that migrated through transwell inserts after incubation with H446 cell- or H1048 cell-derived exosomes, with the numbers of migrated cells indicated in the chart to the right. G. Representative images of tubes formed by HUVECs after incubation with H446 cell- or H1048 cell-derived exosomes; the number of tubes formed is shown in the chart to the right. H. Representative images of mouse aortic rings from which microvessels sprouted after treatment with H446 cell- or H1048 cell-derived exosomes, with the number of sprouted microvessels indicated in the chart to the right. NC, negative control; Exo, exosomes; EXO, exosomes

To determine the function of exosomal miR-141 in SCLC-associated angiogenesis, we treated HUVECs with exosomes derived from SCLC cells to study miR-141 effects on the capacities of cell growth, invasion, migration and tube formation. First, mimics of miR-141 (miR-141) and its NC were transfected into H446 or H1048 cells, which have relatively low expressions of endogenous miR-141 (Supplementary Fig. 1A) and low levels of cell-secreted exosomal miR-141 (Supplementary Fig. 1B). The upregulation of miR-141 in H446 and H1048 cells led to increased levels of miR-141 in exosomes (Fig. 2A-B) with no significant changes in the quantity of released exosomes (Fig. 2C). Vesicles isolated from the CM of SCLC cells were processed for visualization of their appearance by TEM (Fig. 2D), size determination and quantification by nanoparticle tracking technology (Fig. 2C) and protein analysis by western blot (Fig. 2E) to verify the existence of exosomes. Then, we treated HUVECs with the obtained exosomes in which miR-141 levels were upregulated (miR-141-EXO) or the negative control exosomes (NC-EXO) and evaluated their effects on HUVECs. We found that HUVECs incubated with miR-141-EXO grew faster (Supplementary Fig. 2A-B), showed increased migration and invasion (Fig. 2F, Supplementary Fig. 2C-E) and increased tube formation (Fig. 2G). Pretransfection of HUVECs with inhibitors of miR-141 (anti-miR-141) antagonized the promotional effect of miR-141 on cell proliferation, invasion, migration and tube formation (Fig. 2F-G, Supplementary Fig. 2A-E). Moreover, aortic rings that were treated with miR-141-EXO sprouted more and longer microvessels than those treated with NC-EXO, and the increased sprouting was reversed by inhibition of miR-141 (Fig. 2H). Consistent with this observation, CM derived from H446 and H1048 cells transfected with miR-141 mimics also promoted HUVEC proliferation (Supplementary Fig. 3A-B), invasion, migration (Supplementary Fig. 3C) and tube formation (Supplementary Fig. 3D) and increased the number of microvessels sprouting from the aortic rings (Supplementary Fig. 3E). Besides, we treated HUVECs with the plasma of SCLC patients or healthy volunteers to evaluate their effect on the angiogenesis of HUVECs. As shown in Supplementary Fig. 3F-H, compared with the plasma of healthy volunteers, the plasma derived from SCLC patients significantly stimulated the proliferation, invasion, migration and tube formation of HUVECs in vitro.

To further determine whether exosomal miR-141 has direct effects on vascular endothelial cells, we transfected mimics of miR-141 directly into HUVECs and repeated the functional assays. As expected, miR-141 upregulation (Supplementary Fig. 4A) strengthened HUVEC proliferation (Supplementary Fig. 4B), invasion, migration (Supplementary Fig. 4C) and tube formation (Supplementary Fig. 4D). Mouse aortic rings transfected with miR-141 mimics also showed more sprouting microvessels than those transfected with the NC (Supplementary Fig. 4E). To make the result more convincing, another vascular endothelial cell line, EAhy.926, was used to study the impact of miR-141 on angiogenesis. Like the HUVECs, EAhy.926 cells overexpressing miR-141 (Supplementary Fig. 5A) showed enhanced cell proliferation (Supplementary Fig. 5B), invasion, migration (Supplementary Fig. 5C) and tube formation (Supplementary Fig. 5D). Taken together, our results reveal that miR-141 in SCLC cell-secreted exosomes plays critical roles in promoting vascular endothelial cell proliferation, migration, tube formation and angiogenesis in vitro.

### SCLC cell-derived exosomal miR-141 induces neovascularization in vivo

**Fig. 3:**
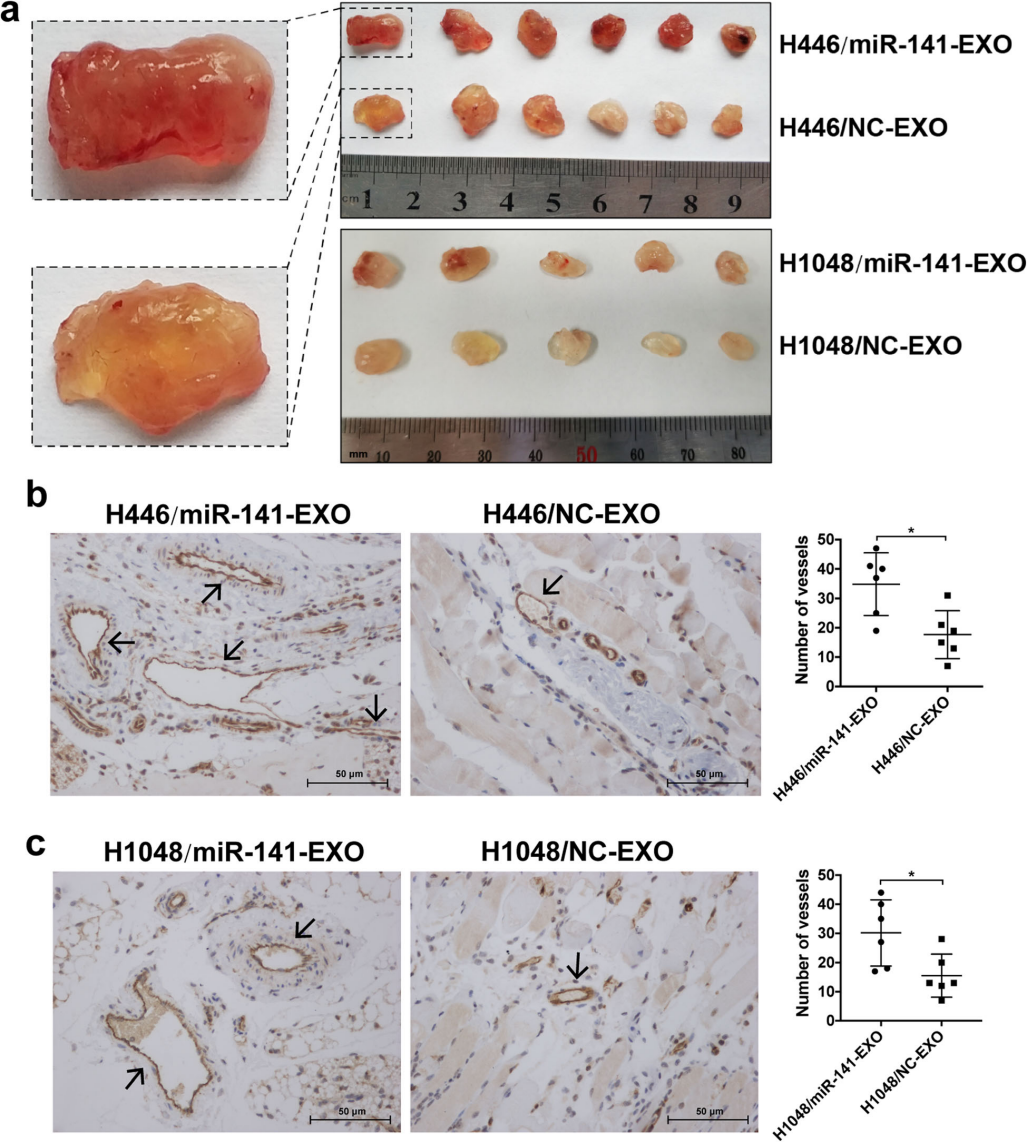
SCLC cell-derived exosomal miR-141 induces neovascularization in vivo. A. Images of Matrigel plugs formed in the mouse axillae after injection with Matrigel which was mixed with H446 cell- or H1048 cell-derived miR-141-EXO or NC-EXO. B, C. Representative images of IHC staining for CD31, with the number of microvessels generated in the Matrigel plugs indicated in the chart to the right

Based on the significant effects of exosomal miR-141 in angiogenesis in vitro, we further explored its function in neovascularization in vivo. Exosomes with miR-141 upregulation were isolated from miR-141-mimic-transfected H446 or H1048 cells. Then, the exosomes were added to Matrigel and the mixtures were injected into the axillae of mice subcutaneously. Formed Matrigel plugs were collected and analyzed 10 days later. The results showed that the Matrigel plugs mixed with miR-141-EXO had more new blood vessels than those mixed with NC-EXO (Fig. 3A). We also conducted IHC staining of CD31 to accurately quantify neovascularization, and the results were consistent with those above, namely, there were more microvessels in the plugs containing miR-141-EXO than in those containing NC-EXO (Fig. 3B-C). This in vivo experiment provides additional evidence of the strong proangiogenic effect of SCLC cell-derived exosomal miR-141.

### miR-141 is transferred from SCLC cells to HUVECs via exosomes

**Fig. 4:**
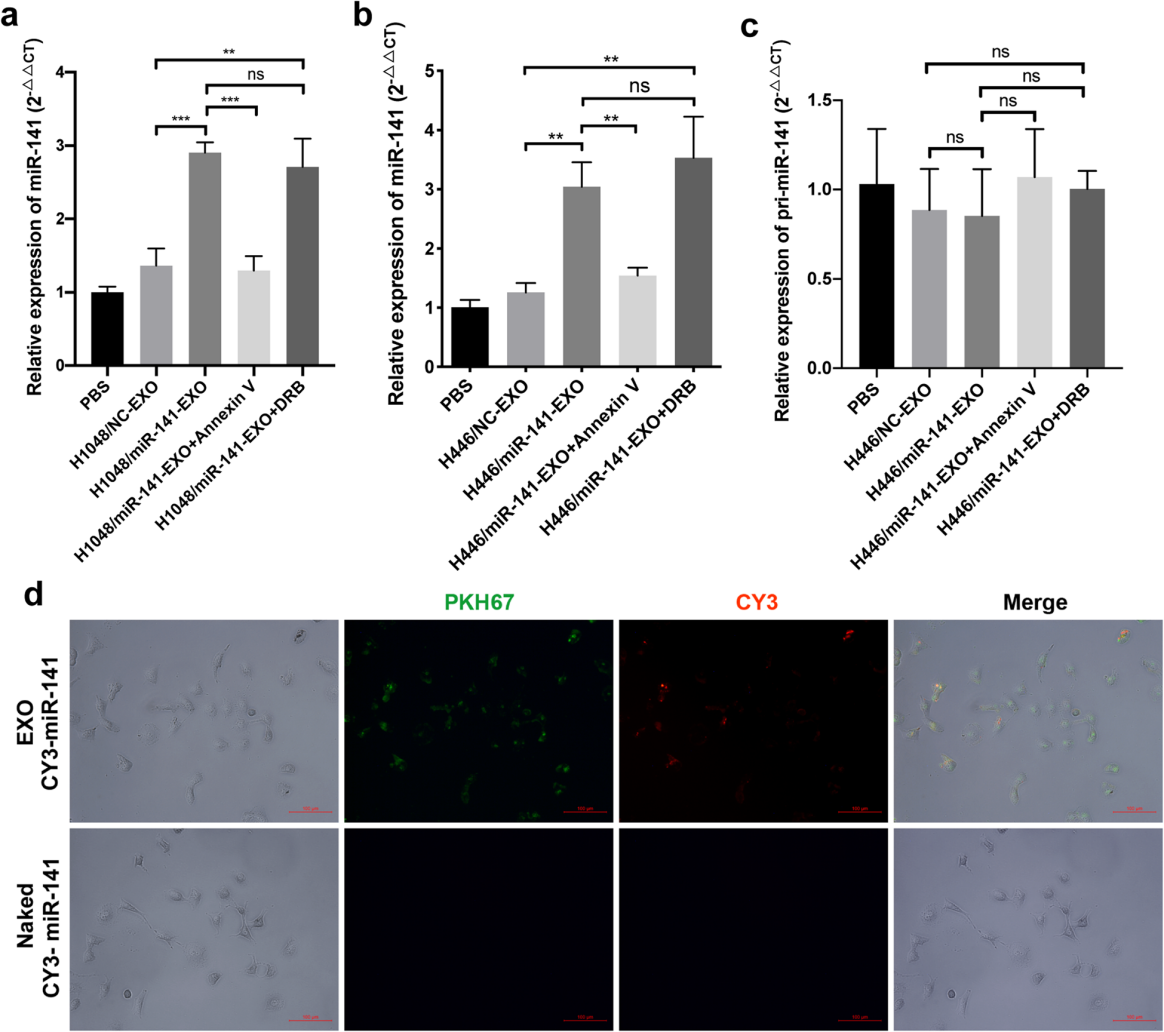
miR-141 is packaged into exosomes and delivered from SCLC cells to HUVECs. A. miR-141 levels in HUVECs increased after incubation with miR-141-EXO derived from H1048 cells, the increase in miR-141 was abrogated by Annexin V pretreatment, and pretreatment of HUVECs with DRB could not reverse the upregulation of miR-141 in HUVECs. B. miR-141 levels in HUVECs increased after incubation with miR-141-EXO derived from H446 cells, the increase in miR-141 was abrogated by Annexin V pretreatment, and pretreatment of HUVECs with DRB could not reverse the upregulation of miR-141 in HUVECs. C. The relative expression of pri-miR-141 in HUVECs did not change after incubation with miR-141-EXO. D. Fluorescence microscopy images of HUVECs after treatment with PKH67-labeled exosomes (green) that contained CY3-miR-141 (red). The mimics of miR-141 with CY3 labels were used as the negative control

We speculated that SCLC-derived exosomal miR-141 was delivered to and internalized by HUVECs to initiate SCLC-related neovascularization. Thus, we conducted a series of experiments to validate this hypothesis. First, we measured miR-141 expression in HUVECs after incubating them with SCLC cell-derived exosomes and found that miR-141 expression in HUVECs was upregulated after incubation with miR-141-EXO (Fig. 4A-B). Pretreatment of exosomes with Annexin V, an inhibitor of exosome absorption, antagonized the increase in miR-141 in HUVECs (Fig. 4A-B), suggesting that the internalization of exosomes in HUVECs leads to the upregulation of miR-141. Additionally, pri-miR-141 expression in HUVECs remained unchanged after incubation with exosomes (Fig. 4C) and pretreatment of HUVECs with an inhibitor of RNA transcription, 5,6-Dichloro-1-β-D-ribofuranosylbenzimidazole (DRB), could not reverse the upregulation of miR-141 in HUVECs after incubation with miR-141-EXO (Fig. 4A-B), indicating that the upregulated miR-141 did not originated from the transcription of pri-miR-141. Furthermore, we prepared PKH67-labeled exosomes that were isolated from Cy3-miR-141 mimic-transfected H446 cells, treated HUVECs with these exosomes, and took photographs of these cells under a fluorescence microscope. As shown in Fig. 4D, miR-141 with red fluorescence was packaged into green exosomes and absorbed by HUVECs via exosomes. Together, these findings demonstrate that miR-141 can be secreted by SCLC cells and delivered to HUVECs via exosomes to mediate its functions in angiogenesis.

### miR-141 promotes angiogenesis by targeting KLF12

**Fig. 5:**
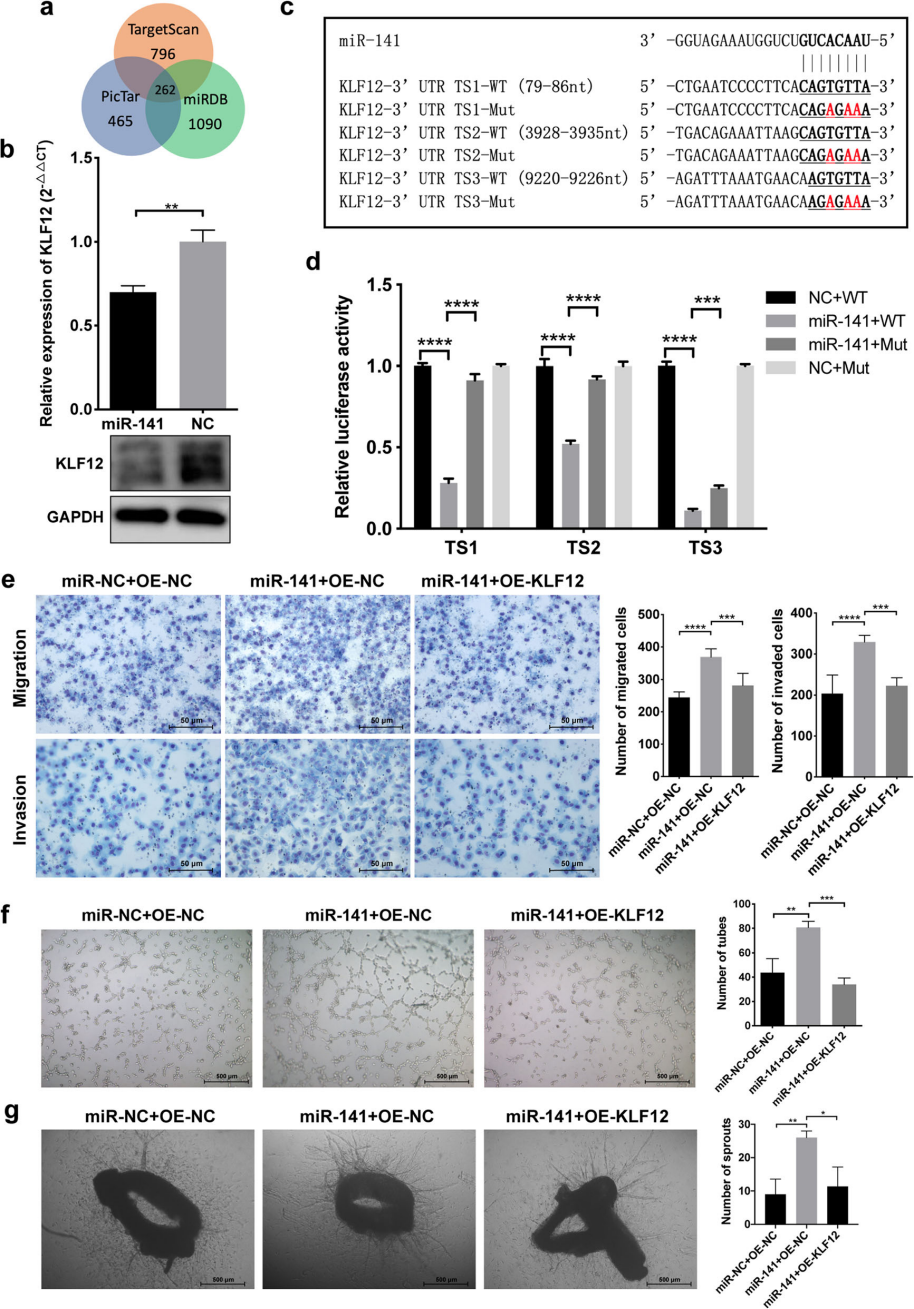
miR-141 promotes angiogenesis by targeting KLF12. A. The number of potential target genes of miR-141 based on the predictions of TargetScan, PicTar and miRDB. B. The mRNA and protein expressions of KLF12 were downregulated in HUVECs after miR-141-mimic transfection. C. The predicted binding sequences of miR-141 in the 3’UTR of KLF12 with the mutated nucleotides marked in red. D. miR-141 inhibits the luciferase activities of reporters containing the wild-type 3’UTR of KLF12, but this activity could be rescued by mutations in seeding sequences. E. Transfection of HUVECs with miR-141 mimics increased the number of migrated or invaded cells, and this was reversed by KLF12 overexpression. F. miR-141 promoted HUVEC tube formation, which was abrogated by the upregulation of KLF12. G. miR-141 stimulated microvessel sprouting from mouse aortic rings, which was abrogated by overexpression of KLF12. TS, target site; WT, wild type; Mut, mutant

We next explored the molecular mechanisms by which secreted miR-141 promoted HUVEC migration and angiogenesis. We searched three databases, including PicTar, miRDB and TargetScan, to predict target genes of miRNAs, and a total of 262 genes common to the three databases were noted as predicted targets of miR-141 (Fig. 5A). The target genes are listed in Supplementary Table 2, with KLF12 being one of the top candidates. Considering that KLF family members have been reported to have various effects on vascular endothelial cells and tumor-associated angiogenesis [29, 33, 34], we speculated that KLF12 might play a critical role in SCLC angiogenesis and that miR-141 promotes neovascularization via KLF12. To confirm our hypothesis, we first detected the level of KLF12 in HUVECs after transfection with the mimics of miR-141. The results showed that mimics of miR-141 reduced the mRNA and protein levels of KLF12 in HUVECs (Fig. 5B), which suggested that KLF12 might be regulated by miR-141. Therefore, we constructed luciferase plasmids containing the wild-type or mutant target sequences of the KLF12 3’UTR (Fig. 5C) for dual-fluorescence assays. As shown in Fig. 5D, the luciferase activities of three reporter plasmids carrying wild-type target sequences were significantly inhibited by miR-141, whereas this inhibition was abrogated when the predicted sequences were mutated (Fig. 5D). These results indicate that KLF12 is directly regulated by miR-141, but whether miR-141 promoted angiogenesis via KLF12 was still unclear.

Then, we synthesized siRNAs against KLF12 to investigate its function in HUVECs. Interestingly, siRNA-mediated knockdown of KLF12 (Supplementary Fig. 6A) significantly promoted HUVEC proliferation (Supplementary Fig. 6B) and increased the number of HUVECs that had invaded or migrated through transwell inserts (Supplementary Fig. 6C), the number of tubes that formed on the Matrigel and the number of microvessels sprouting from aortic rings were also increased after KLF12 knockdown (Supplementary Fig. 6D and 6E). Next, we cotransfected HUVECs with a KLF12 overexpression plasmid that did not contain the KLF12 3’UTR and the miR-141 mimics to determine whether the proangiogenic effects of miR-141 could be reversed by KLF12. The results showed that the enhancements in HUVEC proliferation, invasion, migration and tube formation as well as the increase in the number of sprouting microvessels stimulated by miR-141 were abrogated by KLF12 overexpression (Supplementary Fig. 6F, Fig. 5E-G). Taken together, these data demonstrate that KLF12 is a direct target gene of miR-141 and that miR-141 promotes HUVEC proliferation, migration and angiogenesis by regulating the level of KLF12.

### miR-141 increases the MVD of tumors and support tumor growth in mice

**Fig. 6:**
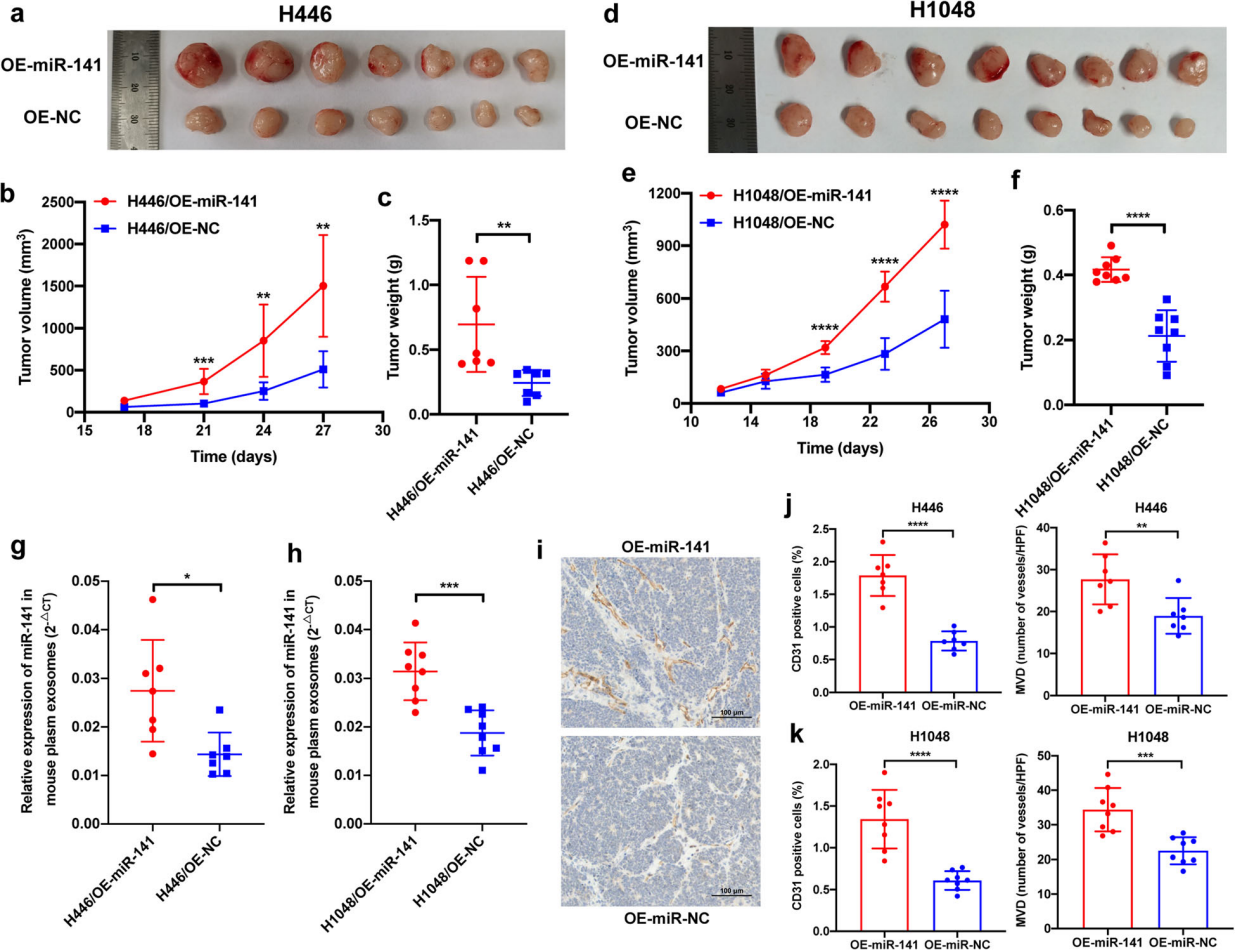
miR-141-overexpressing SCLC tumors grow faster and have increased microvessel densities. A-C. Tumor nodules developed from miR-141-overexpressing H446 cells were larger (B), were heavier (C) and had more external blood vessels (A). D-F. Tumor nodules developed from miR-141-overexpressing H1048 cells were larger (E), were heavier (F) and had more external blood vessels (D). G-H. The level of plasma exosomal miR-141 was higher in mice injected with miR-141-overexpressing H446 or H1048 cells than that in mice injected with negative control cells. I. Representative images of IHC staining of CD31 in paraffin-embedded tumor nodules. J and K. Both the percentage of CD31-positive cells in and the MVD of tumor nodules developed from miR-141-upregulated cells were higher than those developed from negative control cells. HPF, high power field

To further investigate whether miR-141 could promote tumor growth in vivo by inducing neovascularization, we subcutaneously injected miR-141-overexpressing H1048 and H446 cells to the right dorsal flanks of BALB/c nude mice. We found that the tumors that had developed from miR-141-overexpressing SCLC cells were larger, heavier and had more vessels on their surfaces than those that had developed from the NC cells (Fig. 6A-F). To clarify whether this result was attribute to the direct influence of miR-141 on SCLC cells, we conducted CCK8 and transwell assays between H1048/OE-miR-141 and H1048/OE-NC cells or H446/OE-miR-141 and H446/OE-NC cells. As shown in Supplementary Fig. 7A-7D, the proliferation and migration capabilities of both H1048 and H446 cells were not affected by miR-141, which indicated that the increased tumor growth in vivo was not owing to the influence of miR-141 on SCLC cells. Next, we detected the level of miR-141 in mouse plasma exosomes and found that the level of miR-141 in the plasma exosomes derived from the mice with miR-141-overexpressing tumors was significantly increased compared with that of plasma exosomes derived from mice with NC tumors (Fig. 6G and H). We next evaluated the microvessels in the tumor nodules by staining for CD31 and found that both the percentage of CD31-positive cells and the MVD were higher in miR-141-overexpressing tumors than in control tumors (Fig. 6I-K). These results imply that exosomal miR-141 might facilitate SCLC tumor growth in vivo by inducing tumor angiogenesis.

## Discussion

Numerous studies have aimed to dissect the role of angiogenesis, a hallmark of cancer [5], in tumor growth and metastasis, and the various mediators and pathways involved in angiogenesis have been largely characterized, thereby contributing to the development of antiangiogenic therapeutics [35]. miRNAs comprise the most common group of direct or indirect regulators of the angiogenesis associated with different cancer types and thus serve as promising targets for novel antitumor therapies [36]. For example, miR-25-3p induces colorectal cancer angiogenesis by inhibiting the expression of KLF4, thereby downregulating the expression of VEGFR2 in HUVECs [29]. miR-9 promotes the angiogenesis and tumorigenesis of glioma by targeting COL18A1, THBS2, PTCH1 and PHD3 directly [37]. miR-155 induces angiogenesis by regulating VHL in breast cancer and is related to poor outcomes among breast cancer patients [38]. However, studies investigating how miRNAs function in SCLC angiogenesis remain rare. In our present study, we demonstrated that the level of exosomal miR-141 was increased in SCLC patients and that it was essential for SCLC angiogenesis in vitro and in vivo. We observed that the levels of circulating miR-141 were significantly higher in both the plasma and serum of SCLC patients than in those normal volunteers and that the miR-141 expression level was statistically associated with advanced TNM stages, implying the oncogenic role of miR-141 in SCLC. In vitro experiments demonstrated that miR-141 derived from SCLC cells was transported to HUVECs via exosomes and that internalized miR-141 promoted HUVEC proliferation, migration, invasion and tube formation and induced microvessel sprouting. The pro-angiogenic effect of miR-141 was validated in vivo, and tumors of SCLC cells with miR-141 upregulation had an increased MVD and grew faster in mice.

The dysregulation of miR-200 family members, including miR-141, has been demonstrated to be related to various human cancer types, and these miRNAs usually function as tumor suppressors by suppressing EMT and tumor metastasis, inhibiting cancer stem cell (CSC) self-renewal and differentiation and modulating tumor cell growth and apoptosis [39]. In previous studies, the miR-200 family members are regarded as epithelial markers that inhibit the EMT process by directly targeting several EMT-associated genes including ZEB1 and ZEB2 [22, 40], and the upregulation of miR-200 family members is related to the promotion of the mesenchymal-to-epithelial transition (MET) process [41]. In contrast, accumulative data have revealed the oncogenic functions of the miR-200 family, whose members are upregulated in several cancers and are associated with poor clinical outcomes [42, 43], suggesting a dual role for the miR-200 family in tumorigenesis. Indeed, miR-141 has been widely considered a promising biomarker for a variety of cancers, such as non-small cell lung cancer [44], colorectal cancer [45] and prostate cancer [46], and miR-141 levels are correlated with tumor metastatic progression and poor patient prognosis [27, 47–49]. In accordance with these studies, we found that the increase of circulating miR-141 in SCLC patients was significantly associated with advanced TNM stages, highlighting its critical role in SCLC malignancy. Tumor angiogenesis is one process that is induced by miR-141, with increased tumor progression and worsened patient outcomes [27]. However, contradictory reports have indicated that miR-141 can inhibit tumor angiogenesis by targeting multiple downstream genes [25, 26]. With these controversial data, the function of miR-141 in tumor angiogenesis is still largely obscure and the function of miR-141 in SCLC angiogenesis has never been explored. Here, we found that exosomal miR-141 is delivered to HUVECs and promotes SCLC angiogenesis by enhancing HUVEC proliferation, migration, invasion and tube formation and by stimulating the sprouting of new microvessels, which supports the function of miR-141 in angiogenesis.

Further mechanistic explorations showed that the expression of KLF12 was directly regulated by miR-141 and that the proangiogenic effect of miR-141 was rescued by overexpressing KLF12. KLF12 is a member of the Krüppel-like factor (KLF) family, whose members function as transcriptional regulators in a multitude of cancer-relevant processes, including tumor cell proliferation, apoptosis, distant metastasis, tumor inflammation and angiogenesis [50]. The roles of KLF family members, especially KLF2, KLF4 and KLF6, in vascular endothelial cells and angiogenesis have been well studied [33, 34]. In fact, KLF12 has been previously reported to be targeted by miR-141 to strengthen anoikis resistance to facilitate ovarian cancer metastasis [51]. However, the role KLF12 plays in tumor angiogenesis remains obscure. Our study revealed the antiangiogenic effect of KLF12 for the first time. KLF12 knockdown with specific siRNAs significantly promoted HUVEC proliferation, migration, invasion and tube formation, as did miR-141 expression in HUVECs. Moreover, luciferase analysis demonstrated that KLF12 was directly regulated by miR-141, and overexpression of KLF12 in miR-141 mimic-transfected HUVECs abrogated the proangiogenic effect of miR-141, thereby confirming that miR-141 promotes SCLC tumor angiogenesis by targeting KLF12.

## Conclusions

Our results reveal a novel mechanism that underlies the function of exosomal miR-141/KLF12 in SCLC angiogenesis for the first time and provide potential novel targets for anti-angiogenetic therapies for SCLC patients. Our study highlights the critical role of exosomal miRNAs in the SCLC microenvironments and in cell communications which offers novel clues for the future explorations of molecular mechanisms of SCLC malignancy.

## Supplementary information

**Additional file 1 Supplementary Fig. 1.** The endogenous miR-141 level is low in both H446 and H1048 cells and in cell-derived exosomes. A. The relative expression level of endogenous miR-141 in HUVECs and wild-type SCLC cell lines. B. The relative expression level of miR-141 in exosomes isolated from wild-type SCLC cell lines.

**Additional file 2 Supplementary Fig. 2.** Exosomal miR-141 derived from SCLC cells promotes HUVEC proliferation and invasion. A. The proliferation ability of HUVECs incubated with H446 cell-derived exosomes was detected by CCK8 assay. B. The proliferation ability of HUVECs incubated with H1048 cell-derived exosomes was detected by CCK8 assay. C. Representative images of HUVECs that invaded through transwell inserts after incubation with H446 cell- or H1048 cell-derived exosomes. D-E. The number of invaded HUVECs after incubation with H446 cell- or H1048 cell-derived exosomes. EXO, exosomes.

**Additional file 3 Supplementary Fig. 3.** CM from SCLC cells overexpressing miR-141 promotes HUVEC proliferation, migration and tube formation. A. The proliferation ability of HUVECs incubated with H446 cell-derived CM was detected by CCK8 assay. B. The proliferation ability of HUVECs incubated with H1048 cell-derived CM was detected by CCK8 assay. C. Representative images of HUVECs that migrated or invaded through transwell inserts after incubation with CM from H446 or H1048 cells, with the number of migrated or invaded cells indicated in the chart to the right. D. Representative images of tubes formed by HUVECs after incubation with CM from H446 or H1048 cells; the number of tubes formed is shown in the chart to the right; E. Representative images of aortic rings that sprouted microvessels after treatment with CM from H446 or H1048 cells, with the number of sprouted microvessels indicated in the chart to the right. F. The proliferation ability of HUVECs after incubation with the plasma from the SCLC patient or the healthy volunteer. G. Representative images of HUVECs that migrated or invaded through transwell inserts after incubation with the plasma from the SCLC patient or the healthy volunteer, with the number of migrated or invaded cells indicated in the chart to the right. H. Representative images of tubes formed by HUVECs after incubation with the plasma from the SCLC patient or the healthy volunteer, the number of tubes formed is shown in the chart below. CM, culture medium.

**Additional file 4 Supplementary Fig. 4.** The mimics of miR-141 directly promotes the proliferation, migration and tube formation of HUVECs. A. The relative expression level of miR-141 in HUVECs after miR-141-mimic transfection. B. The proliferation ability of HUVECs after transfected with miR-141 mimics or NC. C. Representative images of HUVECs that migrated or invaded through transwell inserts after miR-141-mimic transfection, with the number of migrated or invaded cells indicated in the chart to the right. D. Representative images of tubes formed by HUVECs after miR-141-mimic transfection; the number of tubes formed is shown in the chart to the right. E. Representative images of aortic rings that sprouted microvessels after miR-141-mimic transfection, with the number of sprouted microvessels indicated in the chart to the right. NC, negative control.

**Additional file 5 Supplementary Fig. 5.** Mimics of miR-141 directly promotes the proliferation, migration and tube formation of EAhy.926 endothelial cells. A. The relative expression level of miR-141 in EAhy.926 cells after miR-141-mimic transfection. B. The proliferation ability of EAhy.926 cells after transfected with miR-141 mimics or NC. C. Representative images of EAhy.926 cells that migrated or invaded through transwell inserts after miR-141-mimic transfection, with the number of migrated or invaded cells indicated in the chart to the right. D. Representative images of tubes formed by EAhy.926 cells after miR-141-mimic transfection; the number of tubes formed is shown in the chart to the right. NC, negative control.

**Additional file 6 Supplementary Fig. 6.** Knockdown of KLF12 promotes HUVEC proliferation, migration and tube formation. A. The mRNA and protein levels of KLF12 in HUVECs after transfection with KLF12-specific siRNAs. B. The proliferation ability of HUVECs after inhibition of KLF12 expression. C. The number of HUVECs that migrated or invaded through transwell inserts was increased after inhibition of KLF12 expression. D. The number of tubes formed by HUVECs was increased after transfection with KLF12-specific siRNAs. E. Mouse aortic rings transfected with KLF12-specific siRNAs sprouted more microvessels than those transfected with negative control siRNAs. F. miR-141 promoted HUVEC proliferation, which was abrogated by the upregulation of KLF12.

**Additional file 7 Supplementary Fig. 7.** miR-141 does not influence the proliferation and migration of SCLC cells in vitro. A. The proliferation ability of miR-141-overexpressed H446 cells or control cells. B. The proliferation ability of miR-141-overexpressed H1048 cells or control cells. C. Representative images of miR-141-overexpressed H446 cells or control cells that migrated through transwell inserts, with the number of migrated cells indicated in the chart to the right. D. Representative images of miR-141-overexpressed H1048 cells or control cells that migrated through transwell inserts, with the number of migrated cells indicated in the chart to the right.

**Additional file 8 Supplementary Table 1.** Clinical and pathological characteristics of the patients from whom plasma and serum samples were obtained.

**Additional file 9 Supplementary Table 2.** Target genes of miR-141 predicted by PicTar, miRDB and TargetScan.

## Data Availability

All data generated or analysed during this study are included in this published article and its supplementary information files.

## Abbreviations

SCLC: small cell lung cancer
HUVECs: human umbilical vein vascular endothelial cells
IHC: immunohistochemistry
CM: culture medium
MVD: microvessel density
