# Supplementary figures and images for: Exosomal miR-141 promotes tumor angiogenesis via KLF12 in small cell lung cancer
Source: J Exp Clin Cancer Res. 2020 Sep 21;39:193. doi: 10.1186/s13046-020-01680-1 (PMC7504642; doi:10.1186/s13046-020-01680-1)

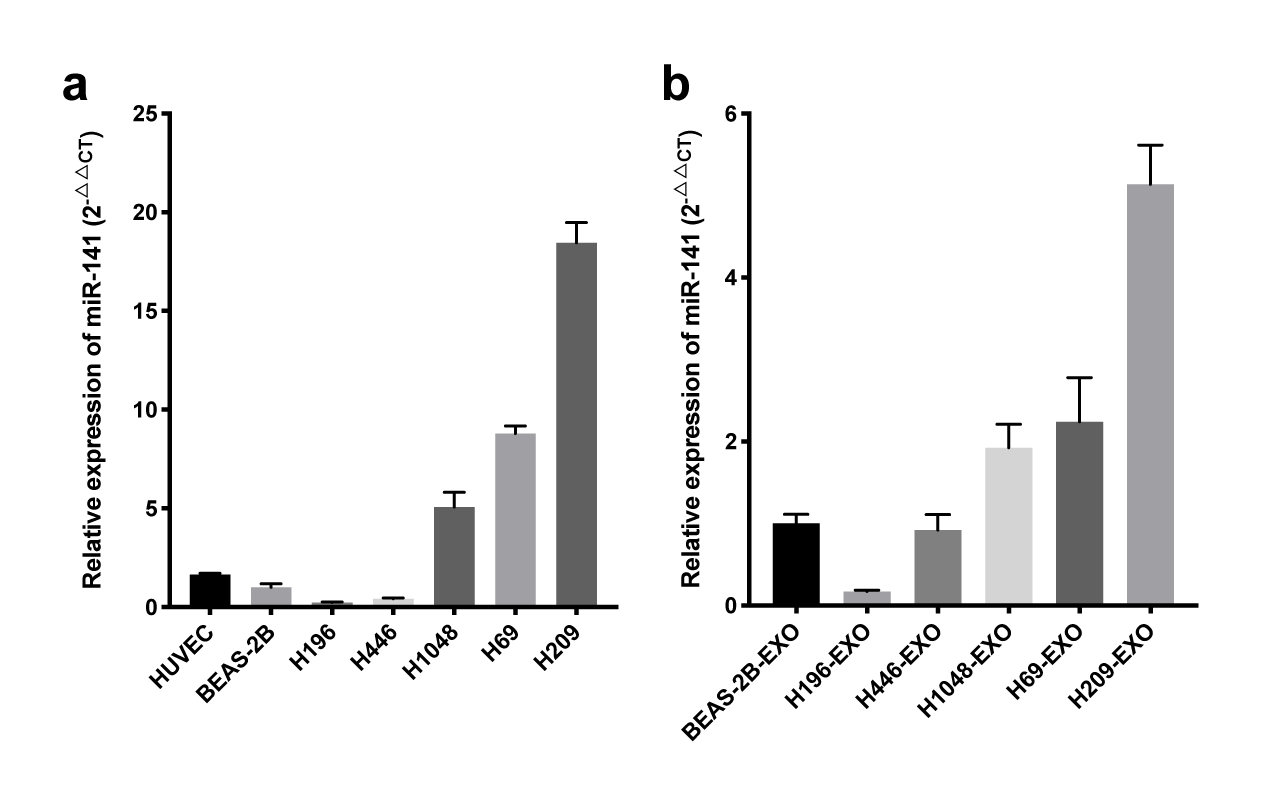

Supplement: Supplementary file 1 — Additional file 1 Supplementary Fig. 1. The endogenous miR-141 level is low in both H446 and H1048 cells and in cell-derived exosomes. A. The relative expression level of endogenous miR-141 in HUVECs and wild-type SCLC cell lines. B. The relative expression level of miR-141 in exosomes isolated from wild-type SCLC cell lines. [file 13046_2020_1680_MOESM1_ESM.tif]

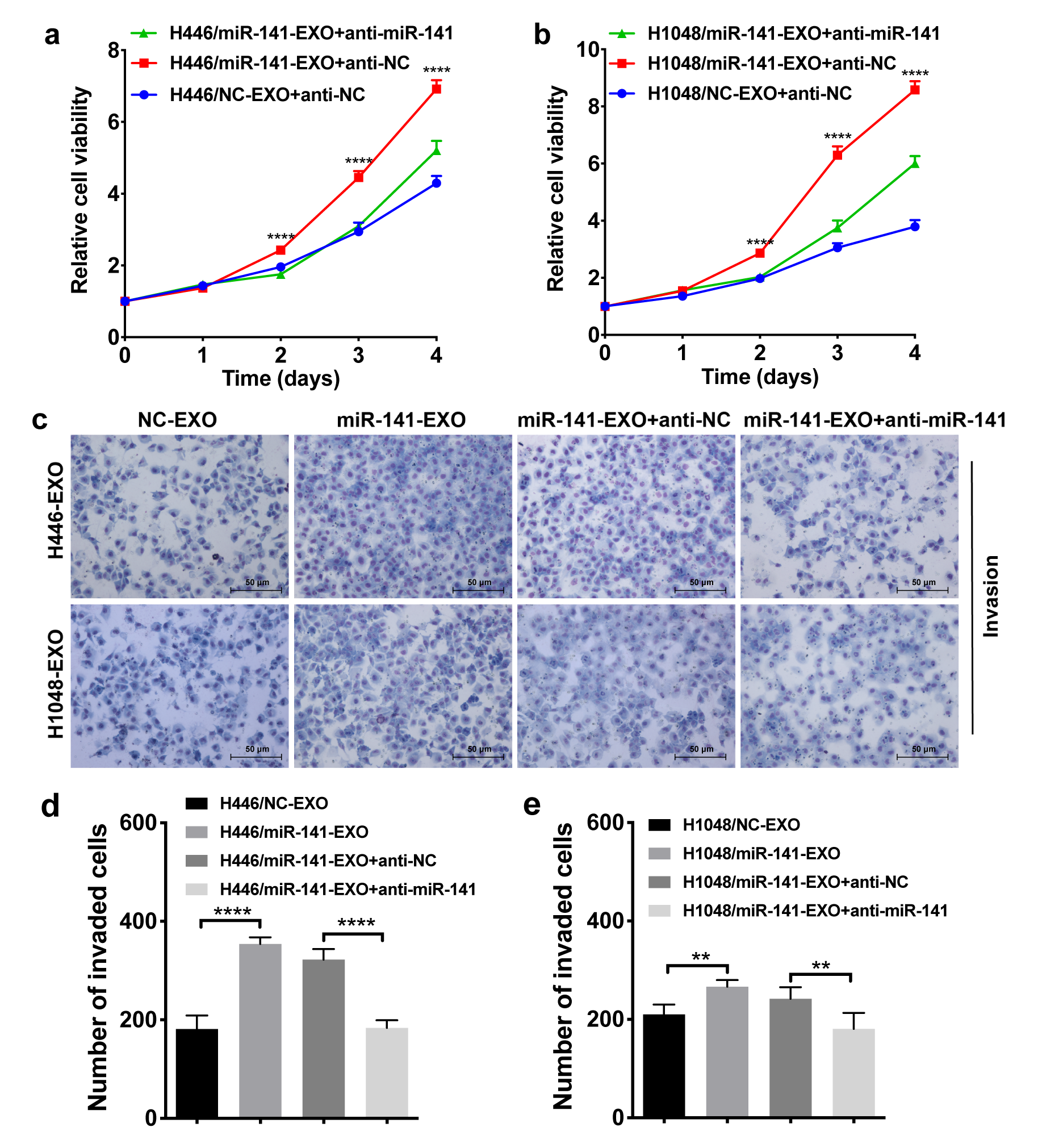

Supplement: Supplementary file 2 — Additional file 2 Supplementary Fig. 2. Exosomal miR-141 derived from SCLC cells promotes HUVEC proliferation and invasion. A. The proliferation ability of HUVECs incubated with H446 cell-derived exosomes was detected by CCK8 assay. B. The proliferation ability of HUVECs incubated with H1048 cell-derived exosomes was detected by CCK8 assay. C. Representative images of HUVECs that invaded through transwell inserts after incubation with H446 cell- or H1048 cell-derived exosomes. D-E. The number of invaded HUVECs after incubation with H446 cell- or H1048 cell-derived exosomes. EXO, exosomes. [file 13046_2020_1680_MOESM2_ESM.tif]

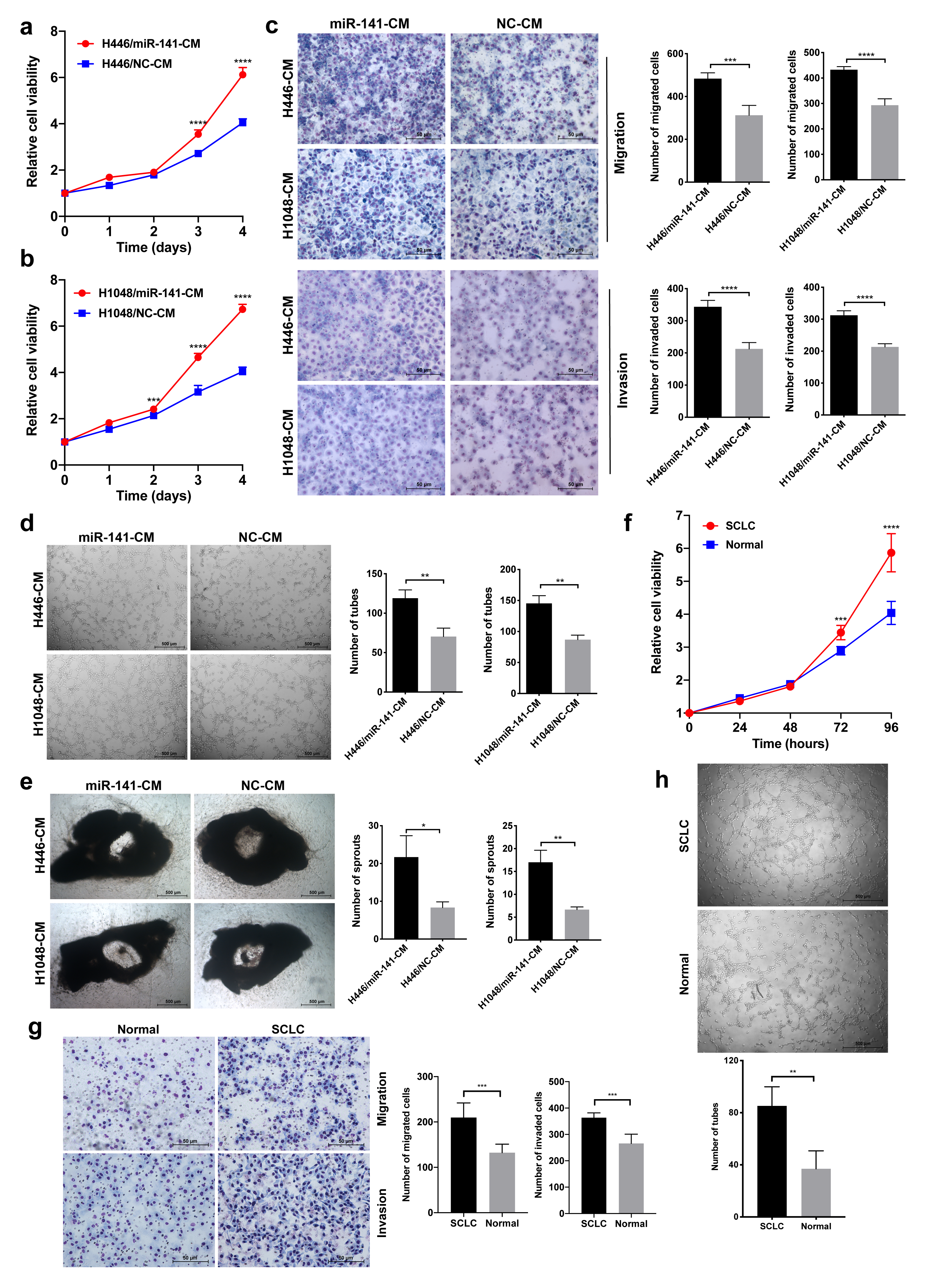

Supplement: Supplementary file 3 — Additional file 3 Supplementary Fig. 3. CM from SCLC cells overexpressing miR-141 promotes HUVEC proliferation, migration and tube formation. A. The proliferation ability of HUVECs incubated with H446 cell-derived CM was detected by CCK8 assay. B. The proliferation ability of HUVECs incubated with H1048 cell-derived CM was detected by CCK8 assay. C. Representative images of HUVECs that migrated or invaded through transwell inserts after incubation with CM from H446 or H1048 cells, with the number of migrated or invaded cells indicated in the chart to the right. D. Representative images of tubes formed by HUVECs after incubation with CM from H446 or H1048 cells; the number of tubes formed is shown in the chart to the right; E. Representative images of aortic rings that sprouted microvessels after treatment with CM from H446 or H1048 cells, with the number of sprouted microvessels indicated in the chart to the right. F. The proliferation ability of HUVECs after incubation with the plasma from the SCLC patient or the healthy volunteer. G. Representative images of HUVECs that migrated or invaded through transwell inserts after incubation with the plasma from the SCLC patient or the healthy volunteer, with the number of migrated or invaded cells indicated in the chart to the right. H. Representative images of tubes formed by HUVECs after incubation with the plasma from the SCLC patient or the healthy volunteer, the number of tubes formed is shown in the chart below. CM, culture medium. [file 13046_2020_1680_MOESM3_ESM.tif]

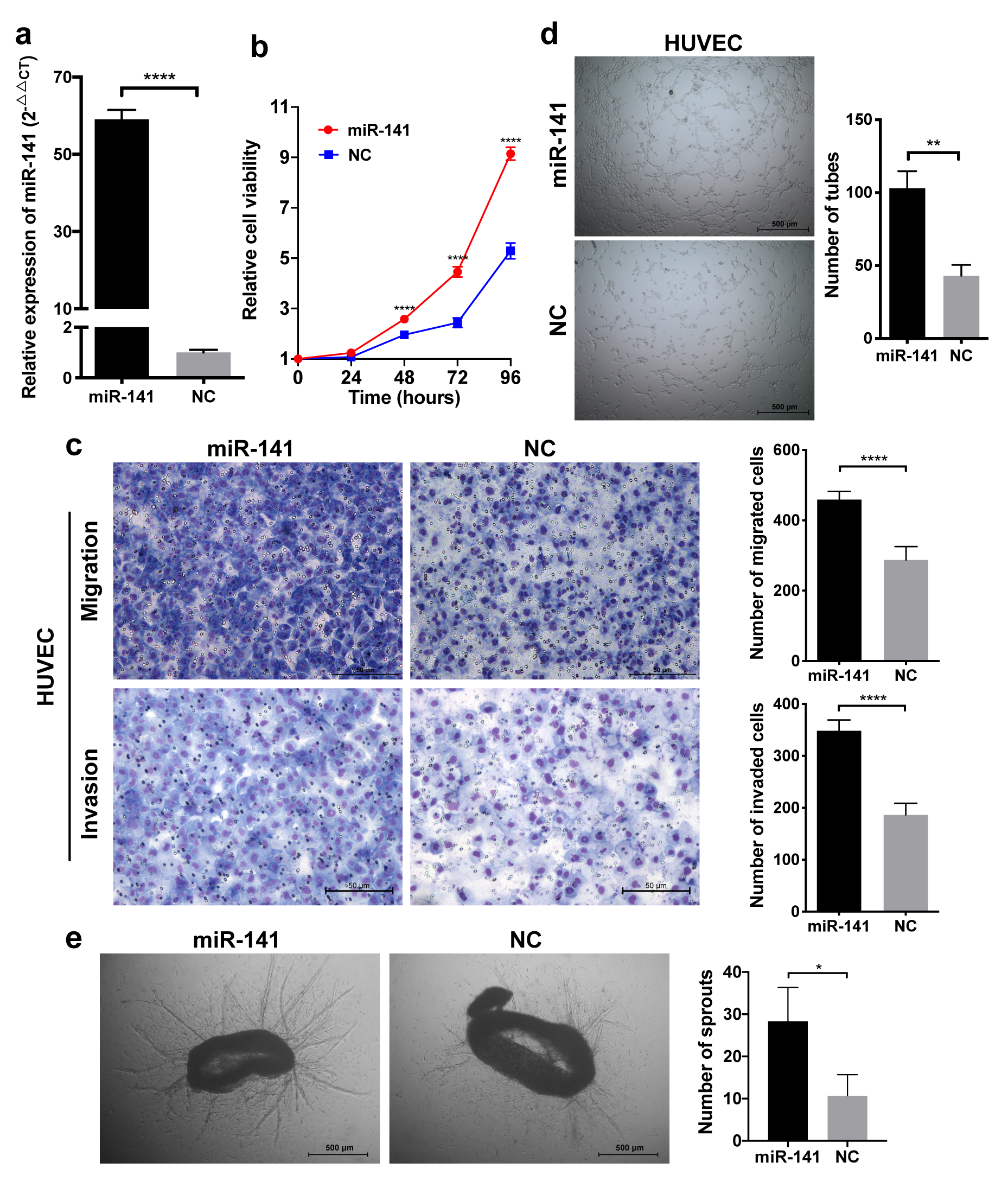

Supplement: Supplementary file 4 — Additional file 4 Supplementary Fig. 4. The mimics of miR-141 directly promotes the proliferation, migration and tube formation of HUVECs. A. The relative expression level of miR-141 in HUVECs after miR-141-mimic transfection. B. The proliferation ability of HUVECs after transfected with miR-141 mimics or NC. C. Representative images of HUVECs that migrated or invaded through transwell inserts after miR-141-mimic transfection, with the number of migrated or invaded cells indicated in the chart to the right. D. Representative images of tubes formed by HUVECs after miR-141-mimic transfection; the number of tubes formed is shown in the chart to the right. E. Representative images of aortic rings that sprouted microvessels after miR-141-mimic transfection, with the number of sprouted microvessels indicated in the chart to the right. NC, negative control. [file 13046_2020_1680_MOESM4_ESM.tif]

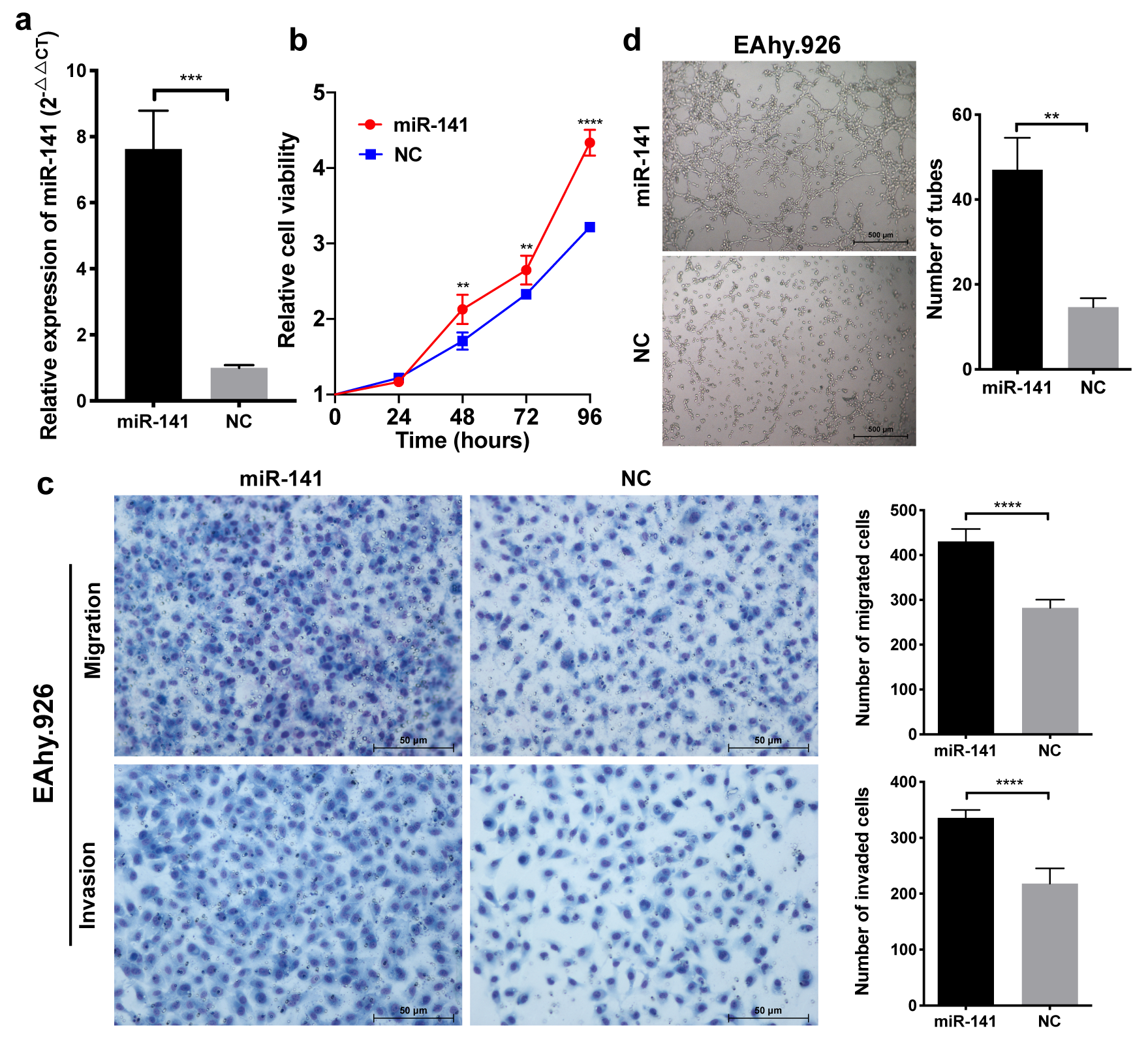

Supplement: Supplementary file 5 — Additional file 5 Supplementary Fig. 5. Mimics of miR-141 directly promotes the proliferation, migration and tube formation of EAhy.926 endothelial cells. A. The relative expression level of miR-141 in EAhy.926 cells after miR-141-mimic transfection. B. The proliferation ability of EAhy.926 cells after transfected with miR-141 mimics or NC. C. Representative images of EAhy.926 cells that migrated or invaded through transwell inserts after miR-141-mimic transfection, with the number of migrated or invaded cells indicated in the chart to the right. D. Representative images of tubes formed by EAhy.926 cells after miR-141-mimic transfection; the number of tubes formed is shown in the chart to the right. NC, negative control. [file 13046_2020_1680_MOESM5_ESM.tif]

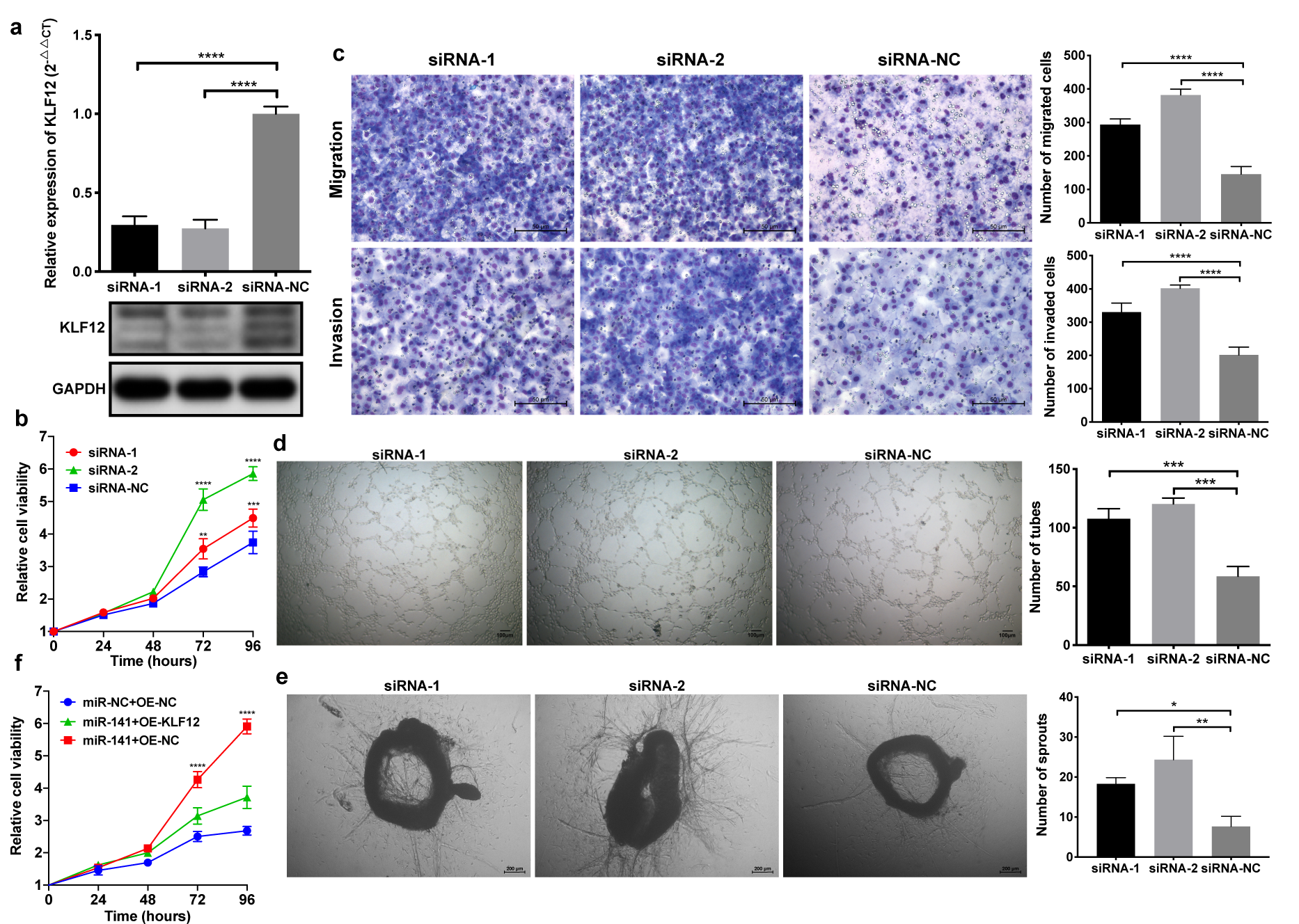

Supplement: Supplementary file 6 — Additional file 6 Supplementary Fig. 6. Knockdown of KLF12 promotes HUVEC proliferation, migration and tube formation. A. The mRNA and protein levels of KLF12 in HUVECs after transfection with KLF12-specific siRNAs. B. The proliferation ability of HUVECs after inhibition of KLF12 expression. C. The number of HUVECs that migrated or invaded through transwell inserts was increased after inhibition of KLF12 expression. D. The number of tubes formed by HUVECs was increased after transfection with KLF12-specific siRNAs. E. Mouse aortic rings transfected with KLF12-specific siRNAs sprouted more microvessels than those transfected with negative control siRNAs. F. miR-141 promoted HUVEC proliferation, which was abrogated by the upregulation of KLF12. [file 13046_2020_1680_MOESM6_ESM.tif]

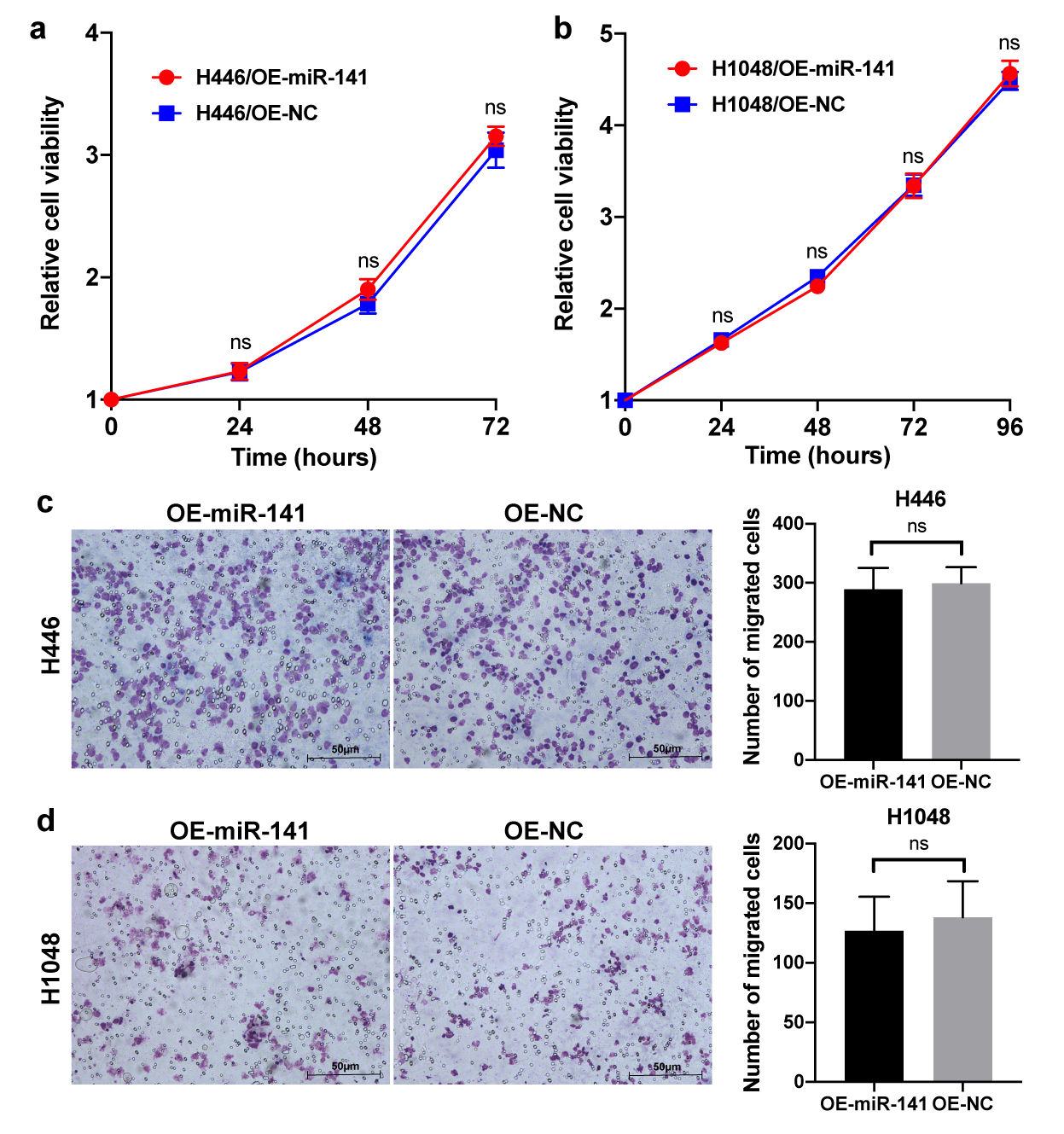

Supplement: Supplementary file 7 — Additional file 7 Supplementary Fig. 7. miR-141 does not influence the proliferation and migration of SCLC cells in vitro. A. The proliferation ability of miR-141-overexpressed H446 cells or control cells. B. The proliferation ability of miR-141-overexpressed H1048 cells or control cells. C. Representative images of miR-141-overexpressed H446 cells or control cells that migrated through transwell inserts, with the number of migrated cells indicated in the chart to the right. D. Representative images of miR-141-overexpressed H1048 cells or control cells that migrated through transwell inserts, with the number of migrated cells indicated in the chart to the right. [file 13046_2020_1680_MOESM7_ESM.tif]
