# Supplementary material for: Exosomal miR-141 promotes tumor angiogenesis via KLF12 in small cell lung cancer
Source: J Exp Clin Cancer Res. 2020 Sep 21;39:193. doi: 10.1186/s13046-020-01680-1 (PMC7504642; doi:10.1186/s13046-020-01680-1)
Supplement: Supplementary file 8 — Additional file 8 Supplementary Table 1. Clinical and pathological characteristics of the patients from whom plasma and serum samples were obtained. [file 13046_2020_1680_MOESM8_ESM.docx]

**Supplementary Table 1. Clinical and pathological characteristics of the patients from whom plasma and serum samples were obtained.**

|  |  | Plasma Samples | | Serum Samples | |
| --- | --- | --- | --- | --- | --- |
|  |  | SCLC (53) | Normal (24) | SCLC (69) | Normal (32) |
| Sex | Female | 19 | 13 | 14 | 10 |
|  | Male | 34 | 11 | 55 | 22 |
| Age | <60 | 28 | 21 | 28 | 14 |
|  | ≥60 | 25 | 3 | 41 | 18 |
| T | T1-T2 | 25 | - | 28 | - |
|  | T3-T4 | 28 | - | 41 | - |
| N | N0-N1 | 17 | - | 10 | - |
|  | N2-N3 | 36 | - | 59 | - |
| M | M0 | 41 | - | 40 | - |
|  | M1 | 12 | - | 29 | - |
| Stage | I | 9 | - | 4 | - |
|  | II | 7 | - | 1 | - |
|  | III | 25 | - | 35 | - |
|  | IV | 12 | - | 29 | - |
| Stage | LD | 32 | - | 35 | - |
|  | ED | 21 | - | 34 | - |

LD, limited disease; ED, extensive disease
